# Supplementary material for: Patterns of Intron Gain and Loss in Fungi
Source: PLoS Biol. 2004 Nov 30;2(12):e422. doi: 10.1371/journal.pbio.0020422 (PMC532390; doi:10.1371/journal.pbio.0020422)
Supplement: Table S1 — Also available at http://genes.mit.edu/NielsenEtAl/. (4.3 MB ZIP). [file pbio.0020422.st001.zip › NielsenEtAl/html/1371.html]

AN0133.1.NCU01612.1.MG03893.1.FG10757.1


```
 CLUSTAL W (1.82) Multiple Sequence Alignments - Introns Inserted


Sequence 1: AN0133.1	769 aa
Sequence 2: FG10757.1	768 aa
Sequence 3: NCU01612.1	853 aa
Sequence 4: MG03893.1	779 aa
Alignment Length: 859 aa
Number Identitical Residues: 472 aa
Alignment Score (without introns) 21381


MG03893.1 	MAEKKGDFNGANKRPSADESDSPQNKRFKGSDATSDGYNPYLAHMNGNGEGDGDG--RSG
NCU01612.1	MASHD-DKGVKRSNMDGNDQDSKRIKRAENGDAAPEKYNPYLAHLNE--DGNGNG--TNG
FG10757.1 	------MSEFMGGKRSSADADESTRKKAKKEGGDDEKYNPYLAHMHDNGDSNGKS--QEP
AN0133.1  	----------MVDRRSDSEDGSRAKRQKMDKSGTDPRDNPYLAHMYADTSSNGNSSSQAD
          	             . .  : ..   ::    ..     ******:  . ..:*..::   

MG03893.1 	SLASLVKGLTRHETTAKQAEKLEDADTNAFTGGSHSQQYFKILKTRRNLPVHQHR~QEFL
NCU01612.1	DFESPLDAFERHKTTAAQAAQAEDSELNPWTGQPHSQRYFDILKVRRDLPVHKQR~QEFL
FG10757.1 	SPDSPLAGMKRQQTTAKQAAKAEDSESNPFTGRPHSQKYFQILQSRRDLPVTKQR~QEFL
AN0133.1  	NKNSAFAKLQRHKTTAAQAQKVEDNEFNPFTNRPYSSKYFSILKTRRDLPVHAQR2DEFL
          	.  * .  : *::*** ** : ** : *.:*. .:*.:**.**: **:***  :* :***

MG03893.1 	DVYHKTQIMVFVGETGSGKTTQIPQYVLYDELPHLNKKMVACTQPRRVAAMSVAQRVADE
NCU01612.1	DMYHNTQILVFVGETGSGKTTQIPQYVLYDELPHTNGKLIACTQPRRVAAMSVAQRVANE
FG10757.1 	DKYHSTQILVFVGETGSGKTTQIPQYVVYDELPHLTGKLIACTQPRRVAAMSVAQRVADE
AN0133.1  	QLYQQSQILVFVGETGSGKTTQIPQFVLLDDLPQTQRKMIACTQPRRVAAMSVAQRVAAE
          	: *:.:**:****************:*: *:**:   *::****************** *

MG03893.1 	LDVELGQEVGYSIRFENKTTPSTILKYMTDGQLLRETIHDPNMTRYSCIIIDEAHERTLA
NCU01612.1	LDVELGQEVGYSIRFENRTGPKTMLKYMTDGQLLRESMHDHDMSRYSCIILDEAHERTLA
FG10757.1 	MDVTLGEEVGYSIRFEDMTGPQTMLKYMTDGMLLREAMHDHEMSRYSCIILDEAHERTLA
AN0133.1  	LDVTLGEEVGYSIRFEDMTSPKTLLKYMTDGMLLREAMNDHELSRYSTIILDEAHERTMS
          	:** **:*********: * *.*:******* ****:::* :::*** **:*******::

MG03893.1 	TDILMALLKQLSLRRSDLKIIIMSATLDAAKFQRYFFEAPILAVPGRTHPVEIFYTAEAE
NCU01612.1	TDILMALLKQIAARRSDLKIIVMSATLDAQKFQSYFYNAPLLAVPGRTHPVEIFYTPEPE
FG10757.1 	TDILMALLKQISMRRPDLKIIIMSATLDAQKFQKYFNDAPLLAVPGRTHPVEIFYTPEPE
AN0133.1  	TDVLMGLLKEVVQRRPDLKIIIMSATLDAQKFQRYFNDAPLLAVPGRTHPVEIFYTPEPE
          	**:**.***::  **.*****:******* *** ** :**:***************.*.*

MG03893.1 	RDYIEASVRTVLQIHATEAEGDILLFLTGEEEIEDVCRKVRLEVDELIRDADCGPMEVYP
NCU01612.1	RDYLEAAVRTVLQIHATEPEGDILLFLTGEEEIEDACRRISLEVDDMIRESDAGPMKVYP
FG10757.1 	RDYVEAAIRTVLQIHASEPEGDILLFLTGEDEIEDACRKISLEADELMREVDAGPLAVYP
AN0133.1  	QDYVEAAIRTVLQIHATEDEGDILLFLTGEEEIEDAARKISLEGDEMIREADAGPLKVYT
          	:**:**::********:* ***********:****..*:: ** *:::*: *.**: **.

MG03893.1 	LYGTLPPQQQQKIFNAAPPPLKKGGRPGRKCIVATNIAETSLTIDGIVYVVDPGFSKQKI
NCU01612.1	LYGTLPPAHQQRIFEKAPEPLRKGGRPGRKVIVSTNIAETSLTIDGIVYVVDPGFSKQKI
FG10757.1 	LYGTLPPHQQQRIFDKAPAPLRKGGRPGRKVIISTNIAETSLTIDGIVYVVDPGFSKQKI
AN0133.1  	LYGSLPPHMQQRIFEPAPPPRRPGGRPGRKVIVSTNIAETSLTIDGIVYVVDPGFSKQKI
          	***:***  **:**: ** * : ******* *::**************************

MG03893.1 	YNARIRVESLLVTPISRASAQQRAGRAGRTRPGKCFRLYTEAAFKKELIEQTYPEILRSN
NCU01612.1	YNPRSRVESLLVSPISKASAQQRAGRAGRTRPGKCFRLYTEKAFKKELIEQTYPEILRSN
FG10757.1 	YNPRIRVESLLVSPISKASAQQRAGRAGRTKPGKCFRLYTEKAFKKELIEQTYPEILRSN
AN0133.1  	YNPRIRVESLLVSPISKASAQQRAGRAGRTRPGKCFRLYTESAFKKELIDQTYPEILRSN
          	**.* *******:***:*************:********** *******:**********

MG03893.1 	LSNTILELKKLGVDDLVHFDFMDPPAPETMMRALEELNYLSCLNDDGDLTDLGHMASEYP
NCU01612.1	LANTVLELKKLGVEDLVHFDLMDPPAPETMMRALEELNYLACLDDDGELTRLGGLASEFP
FG10757.1 	LANTVLELKKLGVEDLVHFDLMDPPAPETMMRALEELNYLACLDDDGELTTLGSMASAFP
AN0133.1  	LSSTVLELKKLGIDDLVHFDLMDPPAPETLMRALEELNYLACLDDDGNLTPLGRLASEFP
          	*:.*:*******::******:********:**********:**:***:** ** :** :*

MG03893.1 	LDPALAVMLVSSPEFYCSNEMLSVTSLLSVPQVFMRPANNRKRADEMKEHFKHMEGDHLT
NCU01612.1	LDPALAVMLISSPEFYCSNEILSIVSLLSVPSIWVRPNNARKRADEMKMQFAHPDGDHLT
FG10757.1 	LDPALAVMLISSPEFYCSNEILSITSLLSVPQIFTRPANNRKRADEMKAQFAHPDGDHLT
AN0133.1  	LDPALAVMLISSPEFYCSNEILSITALLSVPQIFVRPASQRKRADEMKNLFAHPDGDHLT
          	*********:**********:**:.:*****.:: ** . ********  * * :*****

MG03893.1 	LLNAYHAFKSEMAKG-TDMKKWCHEHFLSYRHLSSADNVRAQLKGIMERQDLELVSTPFT
NCU01612.1	LLNAYHAFKGAETNG-DDMKRWCHEHFLSYRHLSSADNVRAQLKRIMETHDISLVSTPFQ
FG10757.1 	LLNAYHAFKGQATSDPNSAKQWCHEHFLSFRHLSSADNVRAQLKRIMETHGLELVSTPFE
AN0133.1  	LLNAYHAFKSPEAQE--NPKQWCHDHFLSLRSLQSADNVRMQLLRIMEREELEMISTSFE
          	*********.  :.   . *:***:**** * *.****** **  *** . :.::**.* 

MG03893.1 	DKNYYTNIRRCLVAGFFMQVALRETSG-KVYHTIKDNQNVMLHPSSVLTTDYEWVLYHEF
NCU01612.1	DKNYYTNIRRALLAGFFMQVAMRESSNSKVYKTVKDDQLVMIHPSTSVTSPYDWVVYNEF
FG10757.1 	DKNYYTNIRRALLAGFFMQVAMKESSGK-LYRTVKDDQAVLIHPSTVLRTEFDWVLYNEF
AN0133.1  	DKKYYENIRRALCAGFFMQVAKKENQGKSMYMTIKDHQNVLLHPSTVLAHDAEWVLYNEF
          	**:** ****.* ******** :*.....:* *:**.* *::***: :    :**:*:**

MG03893.1 	VLTSKQYIRTVTGIRPEWLI~DIAPNYYDLDNFEDDQAKRALKSVVEKMKRREASRGAQG
NCU01612.1	VLTTKQYVRTVTNIRPEWLL0EIAPVYYDLDTFEKGEIKSALTRITDKVKRRQAIKGSGS
FG10757.1 	VLTSKQYIRTCTGIRPEWLL0EIAPTYYDIDSFEQGDVKRSLARAAEKKRRKEAMKAGR-
AN0133.1  	VLTTKNYIRTVTAVKPEWLI0DIAPTYYDISTFPKGDIRSSLLRAAERLSRKEKMRADSG
          	***:*:*:** * ::****: :*** ***:..* ..: : :*   .::  *::  :.  .

MG03893.1 	AS~--SRR----------------------------------------------------
NCU01612.1	AP1DGSGRCEETKDTEKEKPSRPPLVHVTENIARTGATSSAGNSNEKQEKENLKIKKRGA
FG10757.1 	--~---------------------------------------------------------
AN0133.1  	KR~R--------------------------------------------------------
          	                                                            

MG03893.1 	----------------------
NCU01612.1	RRGRRRGGGKDREGKKMVMEGQ
FG10757.1 	----------------------
AN0133.1  	----------------------
          	
```
